# Lip Reading in the Seventeenth Century

**Published:** 1904-09-24

**Authors:** 


### Lip Reading in the Seventeenth Century.

Sir Kenelm Digby gives the following interesting account of an early case of teaching a deaf mute to speak by means of lip reading. The date may be taken as March 1623, when the Prince of Wales, afterwards Charles I., and the Duke of Buckingham, with three attendants, passed through France disguised and undiscovered under the names of John and Thomas Smith, to woo the Infanta at Madrid. At Paris they went to a Court ball where Charles saw the Princess Henrietta Maria, with whom he fell in love and afterwards married. In eleven days after their departure from London they reached Madrid, March 7th, 1623, and surprised everybody by a step so unusual amongst great princes. King Philip received his unexpected guest with the greatest courtesy, but Buckingham's arrogance and the Prince's luke-warmedness in regard to the Infanta nearly led to a war between England and Spain. It is clear from the accompanying narrative that Sir Kenelm Digby was one of the three persons who attended the Prince of Wales on his Spanish journey.

"There was a nobleman of great quality that I knew in Spaine, the younger brother of the Constable of Castile, who was taught to hear the sounds of words with his eyes (if that expression may be permitted). This Spanish Lorde was born deaf, so deaf, that if a gun were shot off close by his ear he could not hear it, and consequently he was dumb: for not being able to hear the sounds of words he could never imitate nor understand them. The loveliness of his face and especially the exceeding life and spiritfulness of his eyes, the comliness of his person and the whole composure of his body throughout were pregnant signs of a well-tempered mind within. And therefore all that knew him lamented much the want of means to cultivate it, and to imbrue it with the notions, which it seemed to be capable of, in regard of itself, had it not been crossed by this unhappy accident, which to remedy Physicians and Chyrurgions had long employed their skill, but all in vain. At the last there was a Priest, who undertook the teaching him to understand others when they spoke, and to speak himself that others might understand him, for which attempt at first he was laughed at, yet after some years he was looked upon as if he had wrought a miracle. In a word after strange patience, constancy and pains he brought the young Lord to speak as distinctly as any man whatsoever: and to understand so perfectly what others said that he would not lose a word in a whole day's conversation. I have often discoursed with the priest whilst I waited upon the Prince of Wales (now our gracious Sovereign) in Spaine, and I doubt not but his Majesty remembereth all I have said of him, and much more: for his Majesty was very curious to observe and enquire into the utmost of it. It is true, one great misbecomingness he was apt to fall into, whilst he spoke: which was an uncertainty in the tone of his voice, for not hearing the sound he made when he spoke, he could not steadily govern the pitch of his voice but it would be sometimes higher and sometimes lower, though for the most part what he delivered together he ended in the same Key as he began it. But when he had once suffered the passage of his voice to close, at the opening it again, chance or the measure of his earnestness to speak or reply gave him his tone, which he was not capable of modulating by such an artifice as is recorded Caius Gracchus used when passion in his orations to the people drove out his voice with too great a vehemency or shrillness. He could discern in another whether he spoke shrill or low, and he would repeat after anybody any hard word whatsoever, which the Prince tried often, not only in English but by making some Welshmen that served his Highness speak words of their language, which he so perfectly ecchoed that I confess I wondered more at that than at all the rest, and his Master himself would acknowledge that the rules of his art reached not to produce that effect with any certainty. And therefore concluded that this in him must spring from other rules he had framed unto himself out of his own attentive observation, which the advantages which nature had justly given him in the sharpness of senses to supply the want of this, endowed him with an ability and sagacity to do beyond any other man that had his hearing. He expressed it surely, in a high measure by his so exact imitation of the Welsh pronunciation: for that tongue (like the Hebrew) employeth much the guttural letters, and the motions of that part which frameth them, cannot be seen or judged by the eye, otherwise than by the effect they may happily make by consent in the other parts of the mouth exposed to view. For the knowledge he had of what they said sprung from his observing the motions they made, so that he could converse currently in the light, though they he talked with whispered never so softly. And I have seen him at the distance of a large chamber's breadth, say words after one, that I, standing close by the speaker, could not hear a syllable of. But if he were in the dark, or if one turned his face out of his sight, he was capable of nothing one said."

